# The Strength of Ti-6AL-4V Investigated Using Micro-Pillars

**DOI:** 10.3390/mi16030293

**Published:** 2025-02-28

**Authors:** Rayan B. M. Ameen, Dilveen W. Mohammed, Yu-Lung Chiu, Ian P. Jones

**Affiliations:** 1 Physics Department, College of Science, The University of Duhok, Duhok 42001, Kurdistan Region, Iraq; dilveen@uod.ac; 2 School of Metallurgy and Materials, The University of Birmingham, Birmingham B15 2TT, UK; y.chiu@bham.ac.uk (Y.-L.C.); i.p.jones@bham.ac.uk (I.P.J.)

**Keywords:** strength for α/β-Ti alloy, micro-mechanical test, nano-indentation, plastic flow

## Abstract

Focused Ion Beam (FIB) has been used to create single α-β colony micro-pillars from a polycrystalline commercial Ti-6Al-4V (Ti-64) sample. Each pillar was selected to have either a single alpha phase, a single beta phase, or two α lamella separated by a thin β phase filet. Then, utilizing a diamond flat tip as a compression platen, uniaxial micro-compression tests were performed on the single crystal α and β pillars as well as a tri-crystal α/β/α pillar using a nano-indenter. Then, utilizing a diamond flat tip as a compression platen, uniaxial micro-compression tests were performed on the single crystal alpha and beta pillars as well as a tri-crystal α/β/α pillar using a nano-indenter. Through the use of Electron Back Scattering Diffraction (EBSD) to choose the crystal orientation along the micro-pillar, three distinct unique slip systems have been selectively triggered by maximizing the Schmid factor for each system. The potential to localize a single crystal volume that can be characterized after deformation is one benefit of the micro-compression approach over traditional mechanical testing. The sample strengths compare well with published data. The mechanical properties of the α-β colonies and the single α and β phases have been compared in order to elucidate the role of the α/β interfaces in determining the critical resolved shear stress.

## 1. Introduction

Among the most common types of commercially available metals are titanium alloys. Half of the titanium that is produced is in α/β titanium alloys, which find extensive usage in engineering. Ti-6Al4V is the most typical example, with the composition shown as a weight percentage (and throughout). For its low density, excellent corrosion resistance, and high temperature mechanical qualities, Ti-64 is a popular choice in many sectors, including aerospace, chemical processing, biomedical, and power generation.

At room temperature, at their equilibrium state, α+β titanium alloys include both the α and β phases, as is evident from their names. They merge the α phase’s strength with the β phase’s ductility. These alloys may have a broad range of mechanical characteristics due to the fact that thermomechanical processing can alter their microstructure.

Typically, three types of microstructures can be identified in Ti (α+β) alloys: lamellar, bimodal, and equiaxed.

Slow or intermediate cooling via the beta-transus temperature may readily produce the lamellar microstructure (furnace cooling or air cooling). During the β phase transformation, the alloy components that stabilize the alpha layer are distributed into the alpha lamellae by diffusion control, while the materials that stabilize the β layer are distributed into the remaining volume. During the β phase, the α plates are formed according to the Burgers’ orientation relationship, which states that the two phases’ closed-packed planes and directions are parallel. Due to low interfacial energy on certain atomic planes, the α phase expands faster along these planes distant from the β grain boundaries. According to the Burgers orientation relationship, the α-phase lamellae have orientations related to the β phase according to the following:

$${\left\{110\right\}}_{\beta }∥\;{\left(0001\right)}_{\alpha },\;{\langle 1\bar{1}1\rangle }_{\beta }\;∥\;{\langle 11\bar{2}0\rangle }_{\alpha }$$

Similarly to the first big beta grains, the following beta-phase areas also share the same crystal orientation. The Burgers orientation relationship (OR) between α and β phases is considered to allow easy slip transmission between the α and β phases because one of the three <a> slip vectors in the α phase is nearly parallel to a <111> slip direction in the β phase.

Understanding the stress–strain behavior of the micro-structural components is crucial for making accurate predictions about the deformation behavior of materials. While the basics of plastic behavior in single-phase titanium alloys have been extensively studied, α/β titanium alloys, which consist of two phases, are more complex.

Chan et al. (1981) [1] conducted compression tests on macroscopic samples of the near-α alloy Ti-8Al-1Mo-1V to characterize the room temperature deformation behavior of individual α/β colonies. For many slip systems, the critical resolved shear stress exhibited a noticeable anisotropy. All orientations except one where prism slip was triggered parallel to the wide face of the α/β contact were found to violate Schmid’s law. The creep and constant strain rate compression of single α/β colony crystals of a Ti-5Al-2.5Sn-0.5Fe (Ti-5-2.5-0.5) near α alloy were examined in macroscopic (4 mm × 4 mm × 8 mm) specimens oriented to activate a single slip system in the α phase by Suri et al. (1999) [2]. Researchers Savage et al. [3] examined the room temperature deformation of Ti-6Al-2Sn-4Zr-2Mo-0.1Si (Ti-6-2-4-2) colony crystals orientated for activation of a single slip system. The crystals were macroscopic (3 mm × 3 mm × 8 mm). The fact that one of the three slip vectors in the α phase, a_1_, is almost perfectly matched (within 0.7°) with a <111> slip direction in the β phase is noticed, which means that (a_1_) may be easily transmitted across the α-β interface. On the other hand, a change of approximately 11.5° is necessary to align the (a_2_) type slip direction in the α phase with the most closely matched <111> slip direction in the β phase. This makes slip transmission more challenging and leads to a noticeably higher critical resolved shear stress. On the other hand, for (a_3_) type slip, there is absolutely no correspondence between the slip systems in the two phases, so substantial hardening could be expected. Higher critical resolved shear stress for some of the slip systems should be caused by the barrier to slide. This would activate additional unanticipated slip systems and lead to a deceptive Schmid factor estimate.

The CRSS values of Ti-64 under different experimental and computational settings have been the subject of many investigations [3,4,5,6,7,8,9,10,11,12,13,14,15,16,17,18,19,20,21]. The CRSS values vary depending on the alloy composition and the testing conditions, hence there is no universal agreement on them. Given the ambiguity around the CRSS values in Ti alloys, it would be appealing to measure these parameters on a single α phase at a certain deformation temperature and using the same test circumstances.

To date, CRSS values have been determined from polycrystalline Ti-64 [2,3,6,9,11,12,16,22,23,24,25] at temperatures ranging from room temperature to 727 °C. Nevertheless, it is not always possible to determine the CRSS values for all slip systems using routine uniaxial tests. Even with high Schmid factors, it can be difficult to generate slip on some systems when the CRSS is considerably lower on other systems. Additionally, numerous commercial metals are not available in single crystal form; they are polycrystalline or multiphase materials, making macrocompression difficult as far as understanding fundamental material behavior is concerned: for instance, for determining the effects of the α/β interfaces in Ti-64. The α/β interfaces within these alloys are considered to hinder slip.

In follow-on work to that of Suri et al. [2], Savage et al. [16,26] measured the CRSSs at room temperature of each of the prismatic <a> and basal <a> slip systems via macro-sample tensile testing of single colonies of Ti-6242Si. A strong anisotropy in the primary creep response of the three $a/3<11\bar{2}0>\{10\bar{1}0\}$ prism slip systems was observed. They demonstrated as well important features of both types of slip transmission through the β phase. Suri et al. [2] and Savage et al. [16,26] used macroscopic polycrystalline two-phase Ti alloy samples rather than small-scale experiments on a localized, confined region, which would be better suited to improve our understanding of the fundamental mechanisms at the level of the individual microstructural constituents.

In order to study the deformation behavior, focused ion beam (FIB) microscopy TEM samples (Transmission Electron Microscopy) may be created from the plastic zone underneath the indenter or indentation, especially with the development of nano-indentation devices for stress–strain behavior determination. The nano-indentation method is often used to ascertain the mechanical characteristics of microscopic volumes due to its ability to attain force resolutions of 1 nano-Newton and displacement resolutions of sub-nanometers.

Specifically, Gong et al. [9,27,28] examined room temperature plastic deformation via the major slip systems in various Ti alloys under constant strain rate compressive loading conditions. The focus of Gong et al.’s [9,27,28] work was to compare the mechanical response and deformation modes in microcantilevers oriented to activate the different slip systems, which can be studied individually, and their CRSSs determined and also how the micro-cantilever width and, therefore, depth, influence the CRSS values.

In Ding et al.’s [6,12,25] associated work, FIB was used to make thin foils for TEM analysis of the dislocation structures obtained. In order to quantify the CRSS values for various slip systems, the micro-cantilever bending experiments require crystal plasticity finite-element simulations. This is performed via a technique that involves modeling and comparison with experimental results.

Table 1 presents reported values of the proof stress for the α/β colonies in Ti-64, Ti-6242, and Ti-5-2.5, under the same compression test conditions and at the same deformation temperature.

In this study, Ti-64 has been subjected to micro-mechanical testing. To find the Schmid factors for the activated slip system, the CRSSs were calculated from the 0.2% proof stress for individual grains (α, β, and α/β/α) using electron back scattering diffraction (EBSD) data and identifying visible slip bands relative to the loading directions. By using micropillar compression, researchers have been able to concentrate in on the micro-mechanical deformation of Ti alloys caused by the two crystal structures’ distinct slip systems. Generally, true stress and strain can be obtained more directly than for a microcantilever by converting load and displacement from measurements of the pillar cross-section and height, although the strains are typically overestimated owing to localized deformation of the top of the pillar which acts as a blunt punch.

## 2. Experimental

An appropriate heat-treatment was developed to achieve a deeper comprehension of the slip systems found in the various titanium phases and the function of the α/β phase boundaries. It is challenging to examine dislocations during the β phase since its diameter is typically about a few hundred nm. Consequently, it was necessary to find a thermal treatment that would produce coarse β lamellae with a width of up to several microns. These lamellae could then be ground into micropillars with either a single β phase or a boundary between the β and α phases. It was determined that initial grain sizes up to 500 μm are necessary because the sizes of the β lamellae are limited by the material’s grain size. Commercially available Ti-64 alloys may have grain sizes as big as centimeters, which can be achieved by the use of vacuum heat treatment.

**Figure 1:**
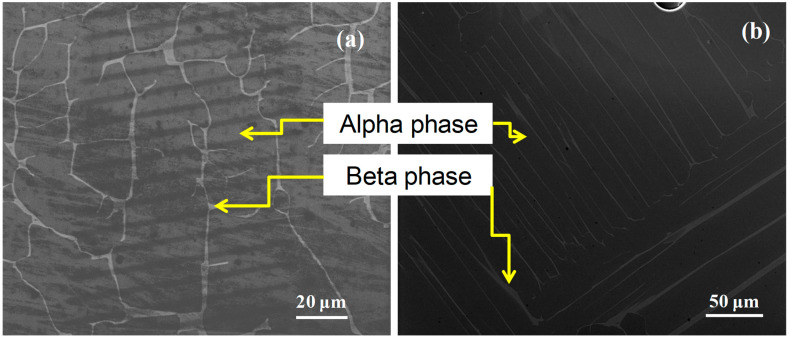
Electron microscopy of a Ti64 furnace after cooling from 1100 °C. In most cases, a microstructure is either equiaxed or lamellar, although in exceptional cases, the latter is more typical (basal plane parallel to polishing surface). In both micrographs, the α phase is dominant, and the β phase is the brightest. What follows makes use of both (**a**,**b**).

The original material was heat treated at 1100 °C for 3 h to produce an equiaxed α/β lamellar microstructure with β phase between the α grains as shown in Figure 1. The α phase lamellae have an orientation that is related to the β phase, according to the Burgers orientation relationship.

The FEI Quanta 3D dual-beam FIB-SEM was used to produce cylindrical micro-pillars from the α, β, and α/β/α alpha colonies in a completely lamellar and equiaxed Ti-64 alloy with an incredibly high starting grain size of 500 μm. The sizes of the pillars varied from 2 to 4 µm, and their aspect ratios were between 2:1 and 3:1.

Cylindrical micro-pillars of 2 μm in diameter were also created to compare the α/β/α proof stresses with those of a single phase β. Given the microstructure, it was not feasible to fabricate single-phase γ pillars with a diameter exceeding 2 μm. Additionally, α and α/β/α pillars measuring 4 μm were made and evaluated.

The crystallographic orientation of the loading axis determines the initial slip system. The Schmid factors for the three a-types, $\langle 1\bar{2}10\rangle$ Burgers vectors ($a_{1},\;a_{2}\;or\;a_{3}$) on basal and prismatic planes and <c+a> for$\;\langle 1\bar{2}13\rangle$ on pyramidal planes, are presented in Table 2.

Cylindrical micro-pillars (α, β, and α/β/α) for each group of specimens were cut from the same colony of alpha grains, in order to keep the crystal orientation constant. Crystal orientation was determined via EBSD, with SEM confirming pillar geometry. Pillars were tilted by 52° to align the Burgers vector with the SEM screen. Slip bands were analyzed by measuring angles between slip traces and the loading axis.

**Figure 2:**
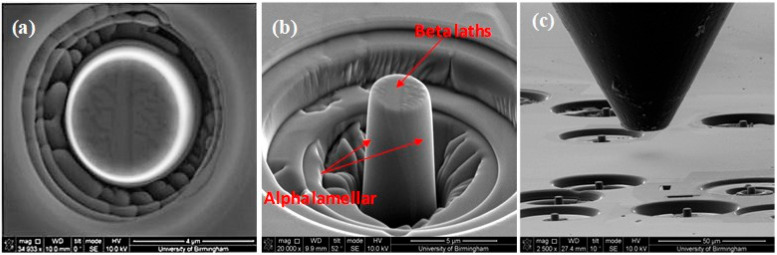
SEM micrographs of a typical micro-pillar fabricated by FIB prior to deformation (**a**) photographed normal to the pillar (**b**) with the pillar tilted 52° to measure its height and (**c**) flat punch and pillars, prior to deformation.

Slip directions were resolved via slip traces orthogonal to the slip plane, consistent with HCP crystallography. The SEM micrographs in Figure 2a,b show a typical 4 μm diameter micro-pillar fabricated by FIB, prior to deformation (Figure 2c).

**Figure 3:**
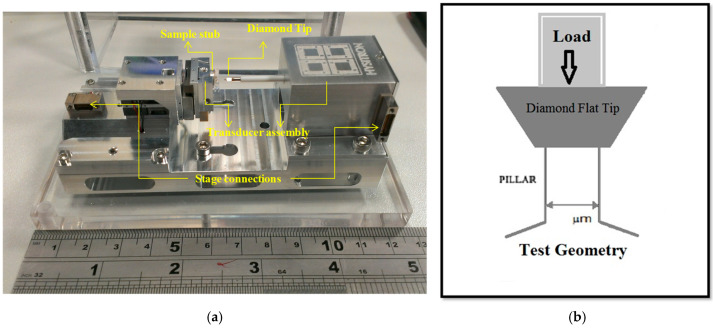
(**a**) The SEM PicoIndenter PI-85, (**b**) a schematic diagram of the flat tip nano-indenter for micro-compression testing.

Tests were performed in situ in a Tescan Mira XM3 FEG-SEM using a Hysitron PI-85 picoindenter equipped with a 20 μm diameter diamond flat punch tip. The uniaxial compression of the micro pillar was at a constant strain rate of $2.5\times 10^{-4}\;s^{-1}$. We used a system which allowed micro-compression in situ (SEM) to ensure proper alignment and gain insight into the deformation process. A schematic diagram of the micro-compression test is shown in Figure 3. It is essential that the top and bottom surfaces of the specimen should be parallel to minimize contact misfit between the sample surface and the compression platen.

This was followed by attaching the specimen using silver DAG paint to the special holder for the PI-85.

In order to determine true stress and true strain, the scanning electron microscope (SEM) is used to measure the micro-initial pillar’s parameters (cross-section and height) prior to loading. After that, a pico-indenter is used to put stress on the micro-pillar, and the SEM is used to measure the distorted dimensions. The applied load and beginning dimensions are used to compute real stress, whereas the deformed and initial heights are used to estimate true strain. Accurate measurement of dimensions and proper equipment calibration are crucial for obtaining precise true stress–strain values.

## 3. Results

**Figure 4:**
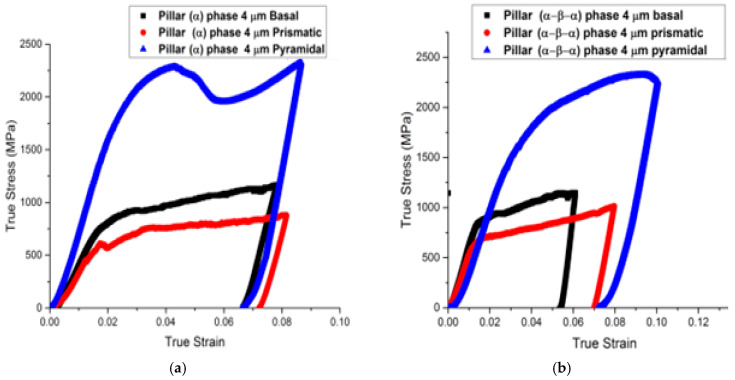
Normal stress–strain curves for (**a**) a single α phase micro-pillar and (**b**) micro-pillars that are alpha/beta/alpha phase, both having a nominal diameter of 4 μm and machined in different crystallographic orientations to activate the <a> prismatic, <a> basal, and <c+a> pyramidal planes, respectively.

Typical true stress–strain curves for microcompression of (α/β/α) and (α) pillars for three different directions are shown in Figure 4.

The α/β/α pillars showed strength disparities when compared to the prismatic, basal, and pyramidal slips. There is a similar relationship between the yield and flow strengths for the basal and prism orientations, but the yield strength is much higher for the pyramidal <c+a> slip. The dominance of single basal and prism slip systems was verified by SEM slip line analysis for these two crystal orientations. In contrast, multiple <c+a> slip occurs on first-order pyramidal planes for the [0001] orientation [24].

A non-linear portion of the stress–strain curve is commonly observed in both macro- and micro-scale compression tests during initial loading stages. This behavior is attributed to surface roughness and geometric imperfections at the contact interfaces. Roughness leads to localized stress concentrations, causing uneven compression as asperities (microscopic peaks) on the specimen surface plastically deform or fracture, gradually conforming to the indenter geometry. Additionally, minor misalignment of the flat punch indenter relative to the micropillar (<1° angular deviation) can induce eccentric loading, generating bending moments or shear stresses that distort the linear elastic response. These effects are particularly pronounced in micropillar compression due to the high surface-area-to-volume ratio and challenges in achieving perfect alignment at microscale resolutions. To standardize comparisons, the proof stress defined as the stress required to induce 0.2% permanent strain in most metals was instead calculated at 5% total strain here, a threshold chosen to bypass early nonlinearities and account for compliance in the testing setup. The resulting proof strains and critical resolved shear stresses (CRSSs) for each slip system are summarized in Table 3.

**Figure 5:**
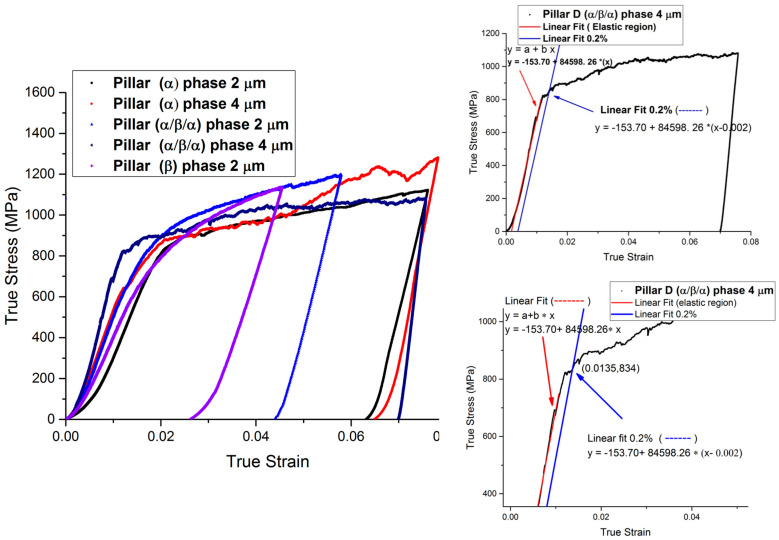
Typical stress–strain curves for micro-pillars with different diameters (2 and 4 μm) oriented to activate <a> basal slip and primary 〈111〉 {101}. The 0.2% strain for pillar (α/β/α) 4 μm is shown in the inset figure.

**Figure 6:**
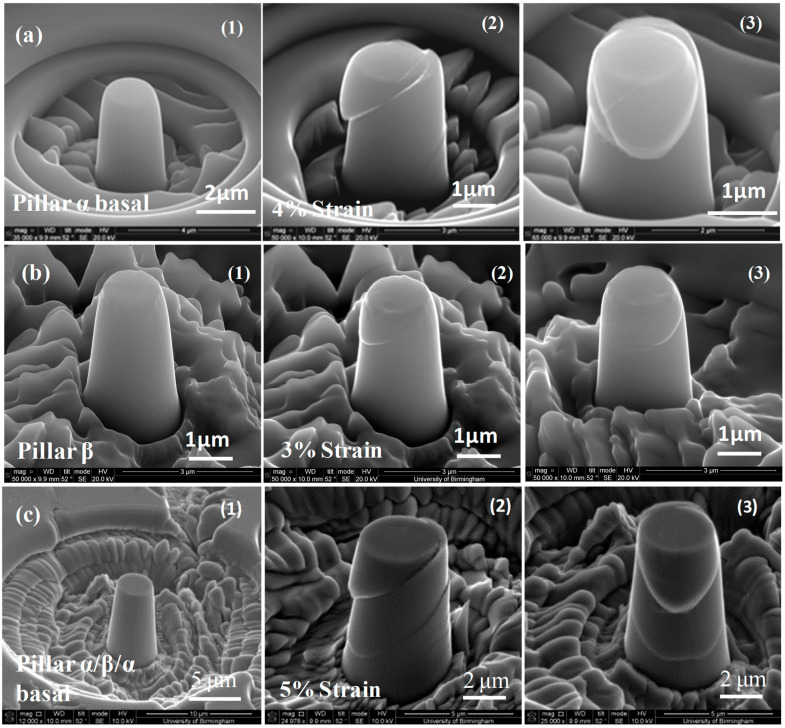
Typical SEM micrographs before and after basal deformation of (**a**) single α phase cylindrical micro-pillars of diameter 2 μm, (**b**) single β phase with a diameter of 2 μm (**c**) (α/β/α) micro-pillars with a diameter of 4 μm. Pillars α, β, and α/β/α are three different specimens (**1**) undeformed (**2**) and (**3**) after deformation (photographs from different directions). Pillar α was strained 4%, pillar β to 3%, and pillar α/β/α to 5%.

**Figure 7:**
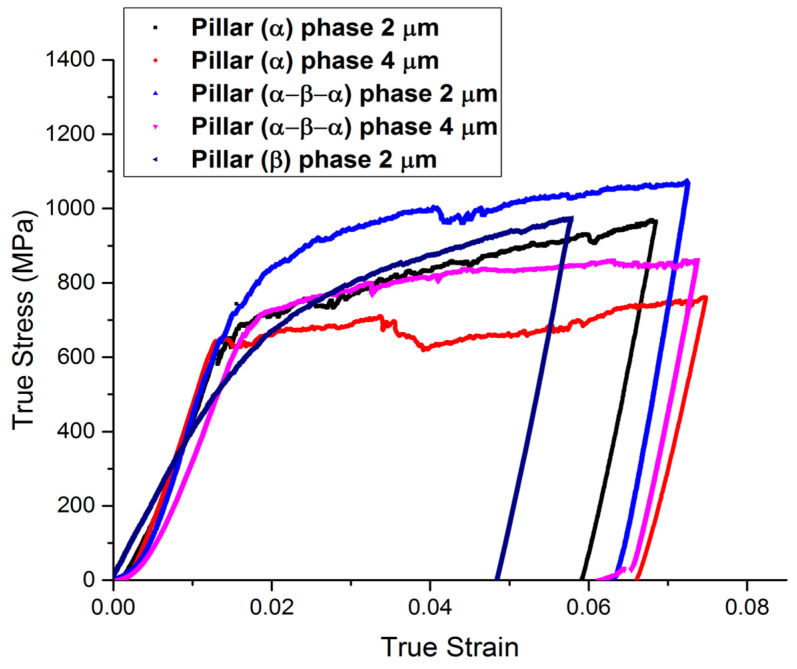
In order to activate <a> prismatic slip and primary 〈111〉 {101}, typical stress–strain curves for micro-pillars with sizes of 2 and 4 μm are prepared.

**Figure 8:**
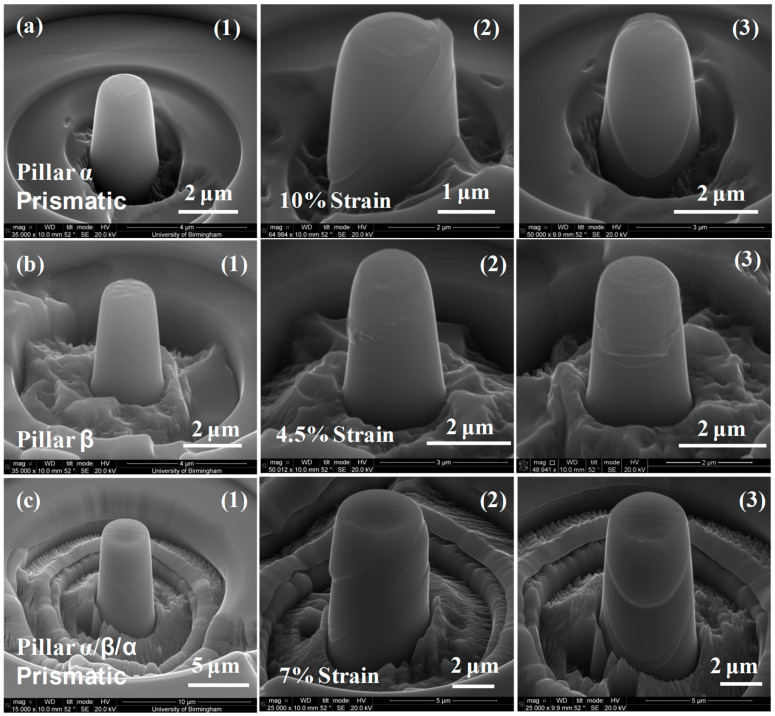
Normal scanning electron micrographs taken before and after prismatic slip deformation of (**a**) 2 μm diameter cylindrical micro-pillars with a single α phase, (**b**) 2 μm diameter micro-pillars with a single β phase, and (**c**) 4 μm diameter (α/β/α) micro-pillars. The three specimens used to create pillars α, β, and α/β/α are (**1**) undistorted, (**2**) deformed, and (**3**) post-deformation (photographs from different directions). The strain on pillar α was 10%, on pillar β it was 4.5%, and on pillar α/β/α it was 7%.

**Figure 9:**
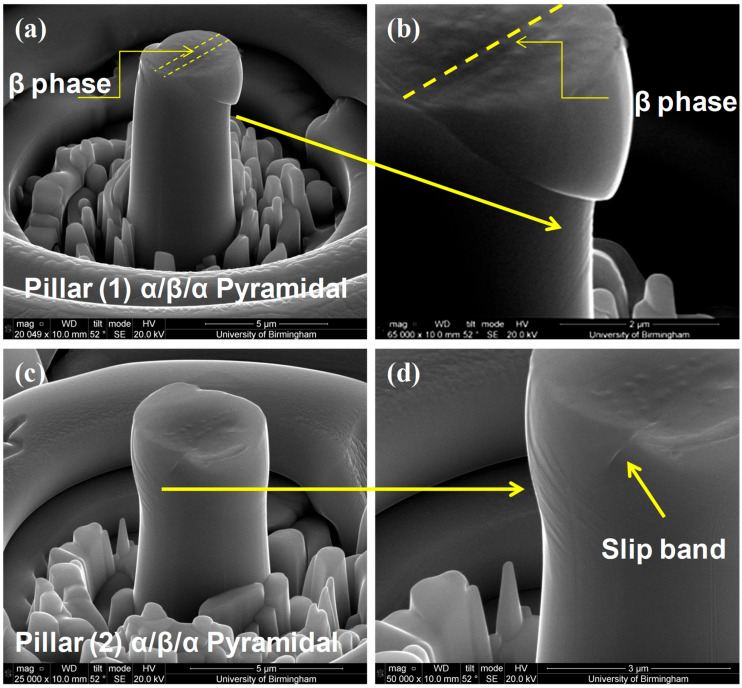
SEM micrographs of pillars (1) and (2) compressed by ~6% strain to activate <c+a> dislocations; (**a**,**c**) showing a clear slip band on the side of the micropillar and shear across α/β/α phases; (**b**,**d**) showing slip traces in addition to the obvious slip bands which are on a different plane.

**Figure 10:**
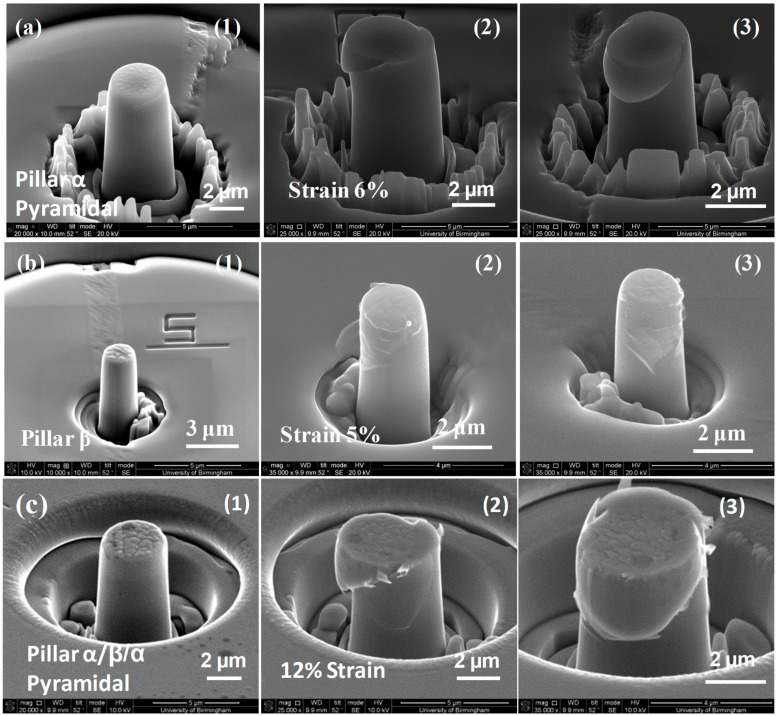
Typical standard scanning electron micrographs taken both before and after distortion of (**a**) 2 μm diameter cylindrical micro-pillars made of a single α phase, (**b**) 2 μm diameter micro-pillars made of a single β phase, and (**c**) 4 μm diameter micro-pillars made of (α/β/α). The three specimens used to create pillars α, β, and α/β/α are (**1**) undeformed, (**2**) deformed, and (**3**) post-deformation (photographs from different directions). A strain of 6% was applied to pillar α, 5% to pillar β, and 12% to pillar α/β/α.

Detailed stress–strain curves (Figure 5, Figure 6, Figure 7, Figure 8 and Figure 9) reveal system-dependent hardening behaviors. For Basal 〈a〉 slip (Figure 5 and Figure 6), the curve exhibits intermittent strain bursts, reflecting stochastic dislocation activity due to limited source availability in micropillars. SEM micrographs corroborate this with isolated slip bands and minimal dislocation entanglement. In contrast, Prismatic 〈a〉 slip (Figure 7 and Figure 8) demonstrates gradual hardening and a lower CRSS (~100 MPa for Ti64, 4 μm pillar), consistent with its role as the primary slip system in HCP Ti alloys. SEM images (Figure 8) reveal planar slip traces and cross-slip events, indicative of dislocation mobility. Pyramidal 〈c+a〉 slip (Figure 9 and Figure 10) displays a pronounced yield drop followed by sustained hardening, aligning with its high CRSS (~350 MPa). Post-deformation micrographs (Figure 10) confirm activation of secondary slip systems and twin nucleation at higher stresses, necessitating complex dislocation dissociation to accommodate 〈c+a〉 glide. The CRSS values in Table 3 reflect this hierarchy: Prismatic 〈a〉 (lowest), Basal 〈a〉 (intermediate, ~15 MPa), and Pyramidal 〈c+a〉 (highest), consistent with Ti64’s HCP deformation mechanics. These results highlight size-dependent stochasticity in Basal 〈a〉 slip, whereas Prismatic 〈a〉’s lower CRSS dominates plasticity in larger volumes.

## 4. Discussion

Three important findings may be drawn from the CRSS data reported in Table 3, which were acquired by the deformation of α/β/α tri-crystals and Ti64 α and β single phase crystals, as will be explained later on: (a) For each loading orientation, the CRSS for the <a> type vectors varies for α single phase crystals and α/β/α tri-crystals. (b) In <a> α/β/α tri-crystals, the CRSS values are larger than for single α phase crystals. (c) In comparison to <a> and <c+a> α/β/α tri-crystals, the CRSS is higher for <c+a> single phase crystals. It is essential to compare specimens of the same size since there is a noticeable size effect (smaller means stronger).

The mechanical properties within the α-β colonies, single α, and β phase have been measured to elucidate the role of the α/β interfaces in determining critical resolved shear stress. Because the critical resolved shear stress in the predicted slip system is larger at the α/β interfaces than at the single α phase, the α/β interfaces within these colonies were thought to impede slide under certain conditions. In spite of the various microstructural components (α and β) having very similar crystallographic orientations, the β laths’ efficacy in limiting slip motion is readily apparent, leading to an increase in strength.

According to Table 3, the β phase is not as powerful as the α phase for <a> slip, but the pillars of α/β/α are stronger than the α phase. Therefore, the β phase prevents dislocation glide at the α/β interfaces, which creates a barrier to slip movement across the colony structure. This resistance ultimately results in a boost in strength [11,22].

An α/β/α CRSS for basal slip is 297 MPa, whereas an α column CRSS for a 4 μm pillar is 268 MPa. According to Table 3, the extra strengthening from the β phase filet is shown by the 29 MPa difference between the α/β/α pillars and a single α phase pillar. This additional strengthening confirms that the α/β interface is effective in impeding slip movement regardless of slip direction.

This confirms what Gong and Wilkinson [9] had studied: that the Ti64 contacts lead to extra strengthening compared to the Ti6Al matrix. The interface is expected to be a considerable slip barrier since the $a_{3}$. The Burgers vector, which was chosen by them in the α phase, does not closely correlate to any of the Burgers vectors in the bcc phase [9]. Based on the closer alignment of the Burgers vectors in the β phase, a reduced strengthening contribution has been seen for the $a_{2}$. Burgers vectors in basal and prismatic slip [9].

While the CRSSs for basal and prismatic slip during a single α phase were lower than those during an α/β/α phase, they were still greater than those during a single β phase. The inclusion of substitutional aluminum makes the α structure harder compared to the β structure, which is more symmetrical.

The slip line observations by in situ SEM performed on the micropillar samples oriented for $a_{2}$ basal and prismatic slip indicate that easy slip transmission through the β phase is probably occurring. The observation of slip via <c+a> pyramidal slip systems implies that the most easily activated initial sources in the α phase are able to continue operating as dislocations transmit through the β phase.

Upon the transmission of 25 $a_{2}$ dislocations through the β lath [2], the accumulated residual interfacial dislocation content may react to form an ($a_{2}$ and $-a_{2}$) matrix dislocation at the entrance and exit α/β interface. The applied stress is likely to drive these $a_{2}$ dislocations into the β phase (referred to as $b_{2}$ dislocations), due to the alpha direction being closely aligned with one of the β directions. This Burgers orientation relationship of the two phase slip directions could explain why shearing of the α/β interface was found [2,3]. Differently sized slip steps were found on the pillar (between the initial dislocation source at the top of the pillar and the released dislocation side on the lower half of the side of the pillar) as shown in Figure 6(c2). For example, Figure 6(c2) shows that there are two clear slip traces on the surface of the right side of the pillar with a large slip step; there are two on the visible left side of the pillar with a slip step smaller than those on the right side. This indicates that there are accumulated residual dislocations near the α/β interfaces, which are suspected to be responsible for the observed higher strength for α/β/α pillars than for other single-phase pillars [3].

Possible causes of strength at the α/β interface have been attributed to the details of the Burgers orientation (OR) between the alpha and β phases as has been extensively reported [1,5,6,7,8,9,10]. The Burgers OR suggests that there should be easy slip transmission across the α/β interface. It is clear that the β laths could provide a different resistance to slip transmission for the three slip directions due to a larger misalignment of the slip directions in the α phase and β phase. One of these three slip directions were always observed to be aligned roughly at the same angle to the broad face of the beta-lath structure, as has been predicted using the invariant line construction [30,31,32,33].

According to studies [6,11,12,25,34], the anisotropy and interface strength are affected by the different Burgers vector lengths in the two phases. The lengths of the Burgers vectors vary between the two phases.

Table 1 compares the proof stresses reported for basal, prismatic, and pyramidal slip systems for this research with previous research under the same conditions. The results of this research show reasonable agreement with a great deal of the previous work in this field given also how much effect heat treatment has on Ti-64. For example, there are similarities between the proof stress for basal and prismatic slip in this study and those reported by Jones and Hutchinson [24] who report CRSS values for <a> slip on the basal and prismatic planes for Ti-64 microcompression. Even though this case is different from Jones and Hutchinson in terms of the absolute magnitude of pyramidal slip [24], their material was very strongly textured. These results are close to those of Ding et al. [6] on the deformation mechanisms in polycrystalline two-phase microcantilever Ti-64 samples.

**Figure 11:**
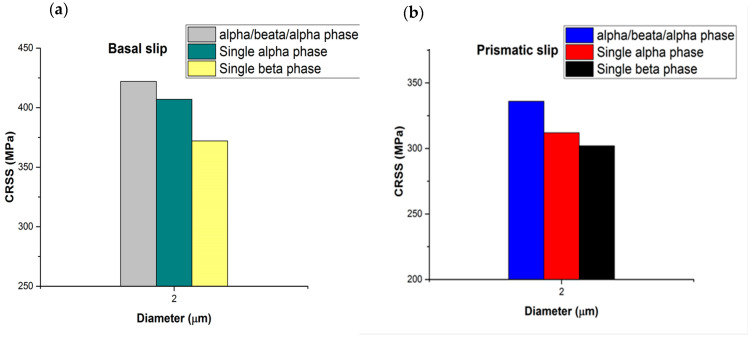
A micro-pillar with a nominal diameter of 2 μm (**a**) for basal slip and (**b**) for prismatic slip is summarized in the CRSS for α/β/α, single α, and β phases.

CRSS is controlled by the plane spacing and the magnitude of the Burgers vector, according to Peierls [35]. The $\left\{1\bar{1}01\right\}$ pyramidal planes have a low packing density and <c+a> is a large Burgers vector. The <c+a> pyramidal dislocations are hard to activate (2–3 times harder than <a> slip due to their large Burgers vector, which is equal to $\left(\sqrt{{\left(a\right)}^{2}+{\left(c\right)}^{2}}=0.553\;nm\right)$. The β phase thickness in the microstructure (lamellar or equiaxed microstructure) is small (≤2 μm) compared to that of the alpha phase even after heat treatment and very slow cooling. Therefore, two pillar diameters were tested to measure the mechanical properties in order to quantify how these microstructural changes contributed to the overall strength of the material. Compared to specimens with a single alpha phase or an α/β/α phase, those with a β phase in the basal or prismatic plane had a lower CRSS value. Consequently, compared to α/β/α crystals and single alpha phases, the CRSS values of the single β phase are lower, as shown in Figure 11.

Comparing the CRSSs for the β phase in Table 3, the CRSS for the β phase in a prismatic oriented colony is smaller than the β phase in the basal and, particularly, pyramidal slip-oriented colonies. The β phase in the basal and pyramidal specimens is closer to straight as they have a lamellar microstructure (see Figure 11a), thus the preparation of the pure single β phase pillar is less complex than the β pillar from the prismatic colony. The β phase for prismatic slip has an equiaxed microstructure (see Figure 2b). Possibly there is some alpha sticking to it.

Comparing the mechanical data for α/β/α and single alpha phase with <c+a> pyramidal slip as summarized in Table 3, we can see that single alpha phase micro-pillars have a larger CRSS value than the α/β/α crystal, which is in contrast to the <a> slip mechanical data. This is because the β phase is much weaker in this situation than the α phase and possibly the interface acts as a source of deformation.

**Figure 12:**
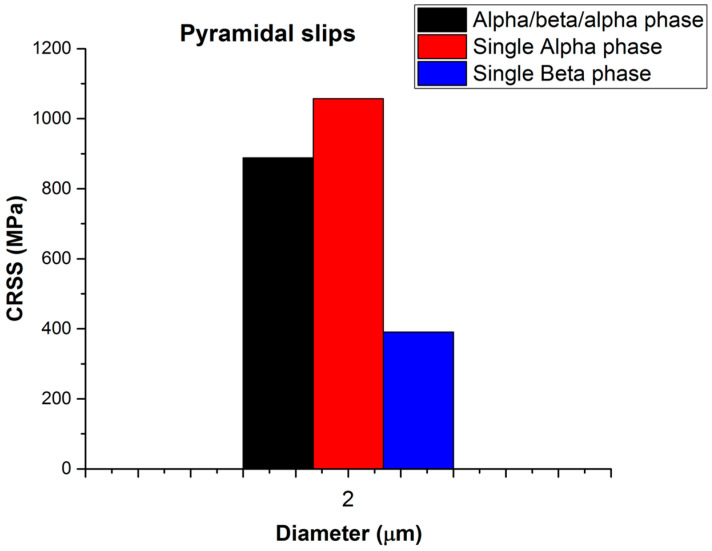
Brief overview of the CRSS for α/β/α, single α, and β phases in a micro-pillar for pyramidal slip with a nominal diameter of 2 μm.

For <a> slip, slip will always start from alpha because the sources are bigger (and, therefore, softer). In the <c+a> situation (see Figure 12) the enormous difference in strength between β and α means that the slip will initiate in the β phase or in the interface and, despite the strengthening effect of both interfaces, the overall strength of the ensemble is less than that of the single alpha phase.

**Figure 13:**
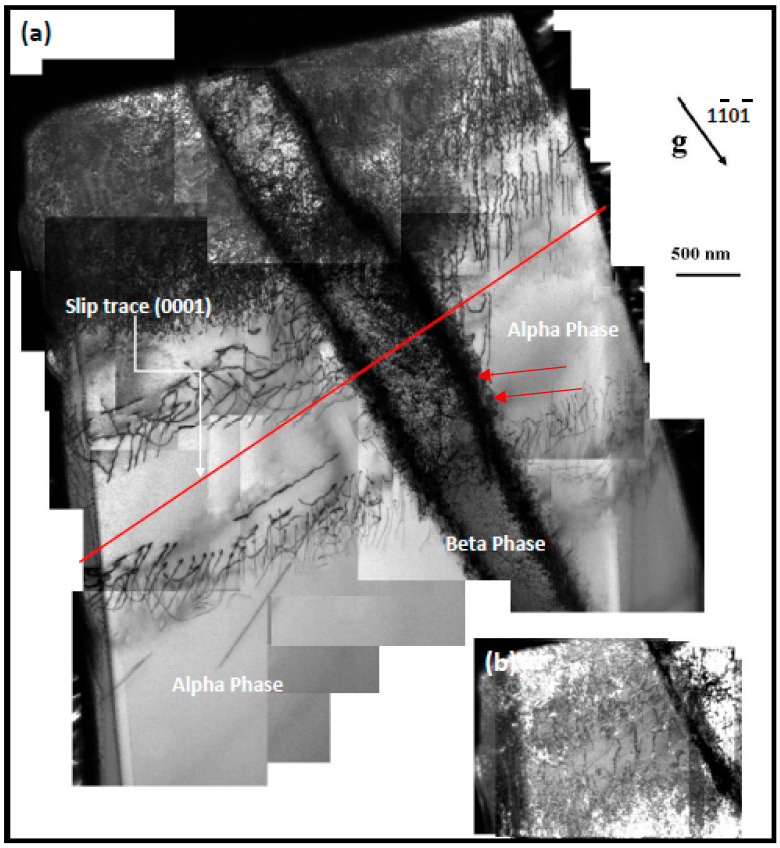
(**a**) A montage of bright field TEM micrographs taken with $g=\left[1\bar{1}0\bar{1}\right]$ near the $\left[1\bar{2}13\right]$ zone axis for (alpha/ β /alpha) micro-pillars with a diameter of 4 μm, which correspond to the same micro-pillars as shown in Figure 6c, (**b**) Left top corner of pillar shown more brightly to facilitate analysis of the dislocations.

Detailed Analysis of Figure 13a presents a bright-field transmission electron microscopy (TEM) montage acquired with diffraction vector $g=[1\bar{1}0\bar{1}]$ near the $\left[1\bar{2}13\right]$ zone axis, capturing dislocation dynamics within a 4 µm-diameter α/β/α Ti-6Al-4V (Ti64) micro-pillar subjected to nanoindentation. The micrograph highlights dense networks of <a>-type dislocations (The Burgerss vector $b=1/3\langle 11\bar{2}0\rangle )$ traversing the hexagonal close-packed (HCP) α-phase matrix. These dislocations originate from localized plastic deformation during indentation, propagating preferentially along basal $\left(\{0001)\}\langle 11\bar{2}0\rangle \right)$ and prismatic $\left(\left\{10\bar{1}0\right\}\left\langle 11\bar{2}0\right\rangle \right)$ slip systems, consistent with the dominant slip activity in HCP titanium alloys under mechanical stress.

Dislocations are notably inhibited at α/β phase borders, resulting in stress-concentrated pile-ups (as illustrated by red arrows in Figure 13a). The body-centered cubic (BCC) β-phase serves as a semi-coherent impediment to dislocation glide owing to crystallographic misalignment, requiring cross-slip or climb processes for circumvention. The curvature of dislocation lines in β-phase areas indicates localized shear stresses surpassing the critical resolved shear stress (CRSS) necessary for slip activation in neighboring α grains. This interaction highlights the involvement of the β-phase in strain hardening through back-stress effects and the growth of forest dislocations.

Figure 13b, which magnifies the upper left corner of the pillar, improves the visibility of dislocations, exposing slip traces that are aligned with basal and prismatic planes. The absence of ⟨c+a⟩ dislocations in the micrograph confirms the restricted activity of pyramidal slip systems at room temperature, highlighting the dominance of <a>-type slip in accommodating plastic strain. The bimodal α/β microstructure influences the micromechanical behavior of Ti64 by concentrating plasticity at phase boundaries, hence enhancing heterogeneous deformation, which is essential for applications demanding fatigue resistance.

These data correspond with classical HCP deformation mechanics while enhancing comprehension of Ti64-specific mechanisms. The direct observation of dislocation-β-phase interactions clarifies uncertainties in slip transfer, emphasizing the significance of phase morphology and crystallographic coherence in enhancing strength-ductility synergy. This work combines TEM crystallography with dislocation theory, establishing a microstructural foundation for the mechanical performance of Ti64 under nanoindentation.

## 5. Conclusions

Micropillar compression is a more easily interpretable method of examining true stress and strain curves than the microcantilever method reported. Micropillar compression is capable of yielding valuable information, especially when the relative CRSSs are determined for individual grains with visible slip bands, based on the Schmid factors on the activated slip system. Generally, true stress and strain can be obtained more easily than for a microcantilever by converting directly load and displacement from measurements of the pillar cross-section and height. It should be in mind, however, that absolute comparisons with macroscopic data remains problematical because the size effect (smaller means stronger) needs to be removed and because the exact shape of the specimen cannot be adequately controlled, neither can the state of the FIB machined surfaces and variation in the microstructure has a disproportionate effect on micro-specimens.

Alpha single crystals have a lower CRSS than α/β/α tri-crystals for basal and prismatic slip. The effectiveness of the α/β interface in restricting slip on basal and prismatic planes is obvious from the mechanical indentation data for compression of α/β/α and single alpha phase micro-pillars. For <c+a> slip, although this is still true, the enormous difference in strength between β and α means that slip will initiate in the β phase and, despite the strengthening effect of both interfaces, the overall strength of the ensemble will be less than that of the single alpha phase.

A strong anisotropy in the critical resolved shear stress for a-type dislocations on basal and prismatic planes in the α/β colony structure has been demonstrated. Generally speaking, the CRSS value for <a> slip on the basal plane for the Ti-64 is higher by 25% than for <a> slip on prismatic planes [34].

In the pyramidal slip systems, CRSSs were found to be significantly higher. This findings in α/β/α tri-crystal Ti-64 alloys [6] corroborate this overarching pattern. An attractive feature of this approach is the high level of quantification achieved for both the single phase and tri-crystal α/β/α phases of CRSS Ti-64.

Differences in the slip mode, changes in the critical resolved shear stress (CRSS) of the β phase, resistance of α/β interfaces to dislocation motion, and density of dislocations and slip systems may explain the observed gap in strength between α phase pillars and α/β/α phase pillars. Further investigation, such as TEM characterization on deformed pillars, is necessary to gain a more comprehensive understanding of these factors and their impact on the observed trends.

## Figures and Tables

**Table 1:** Summary of the proof stress of slip systems at room temperature for Ti alloys.

Materials/Compression Testing	Basal (MPa)	Prismatic (MPa)	Pyramidal <c+a> (MPa)	Ref.
Polycrystal Ti-64	1020	906	1557	[24]
Polycrystal Ti-64	1180	779	-	[29]
Polycrystal Ti-64	-	960	2587	[6,12]
Polycrystal Ti-6242	824	-	-	[3,16,22]
Polycrystal Ti-52	-	755	-	[2]

**Table 2:** α and β phase Schmid factors for loading directions used. Bold signifies operative slip system.

Favored Slip System	Stress Axis	Slip Systems in the α Phase	Schmid Factors in the α Phase	$\mathrm{Schmid}\;\mathrm{Factors}\;\mathrm{for}\;\mathrm{Primary}\;\langle 111\rangle \left\{101\right\}$
Basal	$\left[\bar{1}5\bar{4}6\right]$	$a_{1},a_{2},a_{3\;}$ Basal	0.1, **0.47**, 0.37	**0.49**
		$a_{1},a_{2},a_{3\;}$ Prismatic	0.08, 0.13, 0.2	
		$\langle 11\bar{2}3\rangle$ Pyramidal	0.25, 0.22, 0.21	
Prismatic	$\left[01\bar{1}0\right]$	$a_{1},a_{2},a_{3\;}$ Basal	0.0, 0.0, 0.0	**0.44**
		$a_{1},a_{2},a_{3\;}$ Prismatic	0.0, **0.43**, **0.43**	
		$\langle 11\bar{2}3\rangle$ Pyramidal	0.44, 0.41, 0.33	
Pyramidal	$\left[0001\right]$	$a_{1},a_{2},a_{3\;}$ Basal	0.0, 0.0, 0.0	**0.47**
		$a_{1},a_{2},a_{3\;}$ Prismatic	0.0, 0.0, 0.0	
		$\langle 11\bar{2}3\rangle$ Pyramidal	**0.451**	

**Table 3:** Mechanical data for compression of α, β and α/β/α micropillars, where pillars 1, 2 and 3 are relatively similar with regard to pillar size and 1:2 aspect ratio and $\sigma_{{}^{\circ}}$ is the stress at 5% strain.

Orientations	Phases	Diameter	Pillar 1		Pillar 2		Pillar 3		Average $\;\sigma_{{}^{\circ}}$ (MPa)	Average CRSS (MPa)
			$\sigma_{{}^{\circ}}$	CRSS (MPa)	$\sigma_{{}^{\circ}}$	CRSS (MPa)	$\sigma_{{}^{\circ}}$	CRSS (MPa)		
<a> on basal plane	Pillar (α)	2 μm	872	410	854	402	869	408	864	407± 8
		4 μm	783	368	775	364	780	365	780	366 ± 10
	Pillar (β)	2 μm	767	375	752	369	-	-	759	372 ± 8
	Pillar (α/β/α)	2 μm	905	425	895	420	891	418	899	422 ± 5
		4 μm	807	379	834	391	831	390	824	388 ± 15
<a> on prismatic plane	Pillar (α)	2 μm	711	306	724	311	742	319	726	312 ± 10
		4 μm	610	263	641	276	-	-	623	268 ± 10
	Pillar (β)	2 μm	694	305	675	297	-	-	685	301 ± 10
	Pillar (α/β/α)	2 μm	762	328	802	344	780	335	781	336 ± 15
		4 μm	685	294	687	295	702	302	690	297 ± 7
<c+a> on the first order pyramidal plane	Pillar (α)	2 μm	2293	1034	2337	1053	2407	1085	2339	1055 ± 15
		4 μm	1972	889	1962	885	1925	868	1951	880 ± 20
	Pillar (β)	2 μm	907	426	835	392	860	404	832	391 ± 15
	Pillar (α/β/α)	2 μm	1950	879	1988	896	-	-	1966	887 ± 10
		4 μm	1736	783	1715	773	1720	776	1729	777 ± 10

## Data Availability

The data presented in this study are available on request from the corresponding author.
